# Supplementary figures and images for: Resveratrol Reduces COMPopathy in Mice Through Activation of Autophagy
Source: JBMR Plus. 2021 Jan 22;5(3):e10456. doi: 10.1002/jbm4.10456 (PMC7990140; doi:10.1002/jbm4.10456)

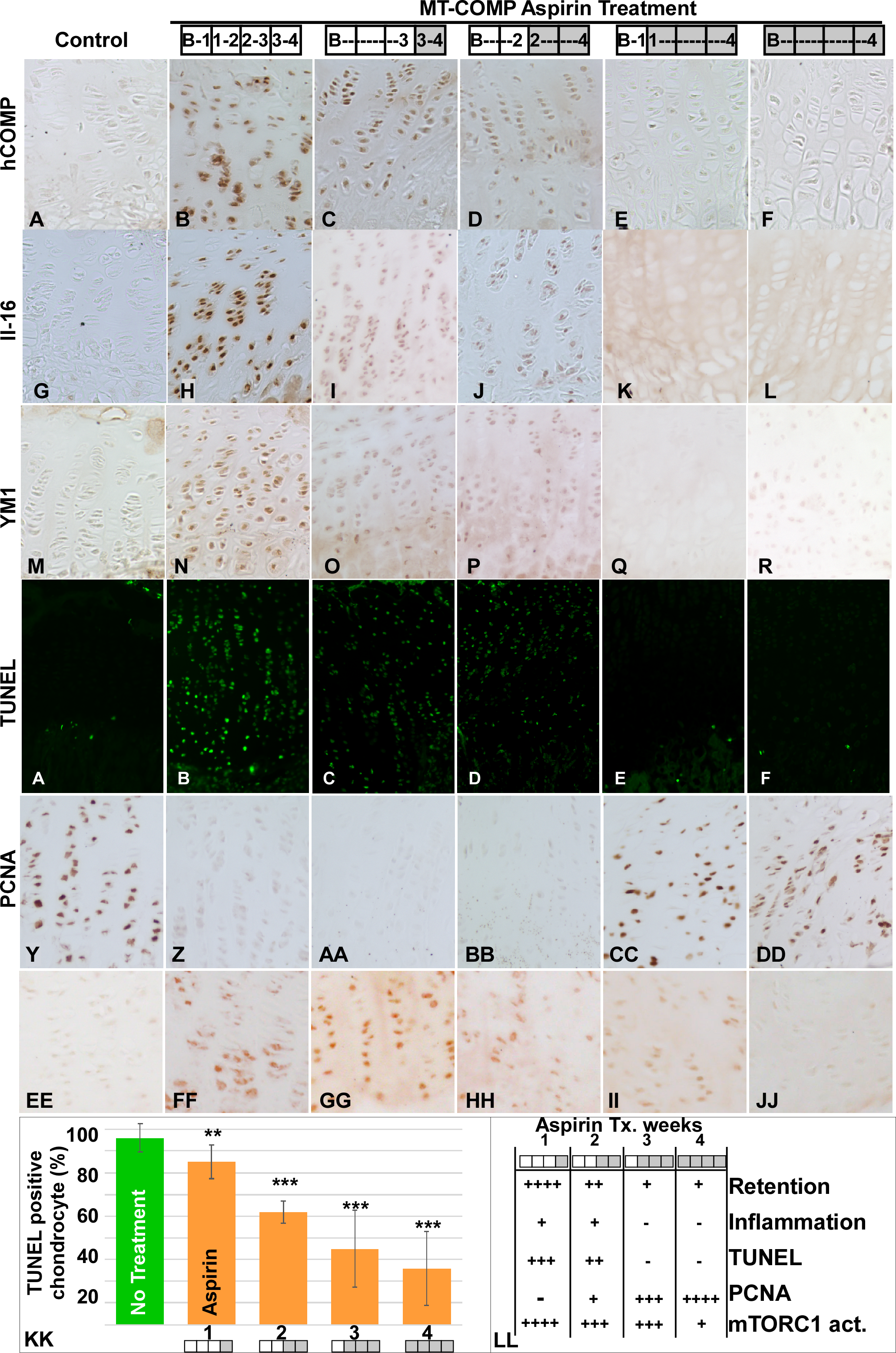

Supplement: Supplementary file 1 — Supplementary Fig. S1. Aspirin treatment window. Treatments started at 1 or 2 or 3 weeks after birth and stopped at 4 weeks and evaluated for mutant‐COMP pathology. Each treatment group included 10 mice. Aspirin treatments are shown in the shaded boxes. Treatments starting at birth or 1 week of age produced the best therapeutic outcomes (K, L), later therapy mitigated but did not eliminate the disease progression (C). Control (C57BL\6) (A, G, M, S, Y, EE) and MT‐COMP with no treatment (B, H, N, T, Z, FF) resveratrol treatment for 1 (C, I, O, U, AA, GG), 2 (D, J, P, V, BB, HH), 3 (E, K, Q, W, CC, II) or 4 (F, L, R, X, DD, JJ) weeks and growth plates at 4 weeks were evaluated for human COMP retention, IL‐16 and YM1, TUNEL (cell death), PCNA and mTORC1 signaling. Quantification of TUNEL positive chondrocytes for different treatment periods are shown in KK. Bars = means with standard deviation. Aspirin treatment decreased intracellular MT‐COMP after 2 weeks of treatment (D) and decreased in IL‐16 and YM1 inflammation after 1 week of treatment (I, O) compared to (H, N). Aspirin decreased cell death after 2 weeks of treatment (U), mTORC1 signaling after 3 weeks of treatment (JJ) and increased proliferation after 3 weeks of treatment (CC). The relative change of MT‐COMP pathology on treatment period is shown in LL. [file JBM4-5-e10456-s002.tif]

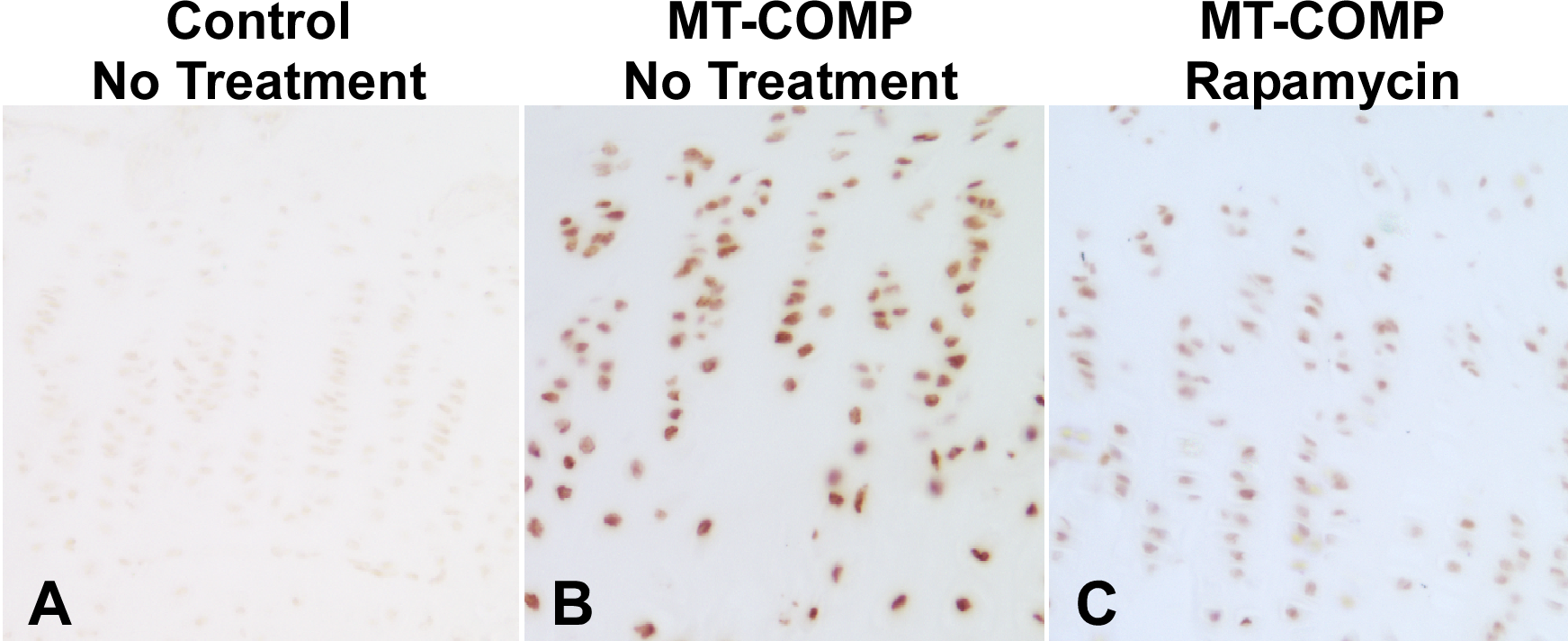

Supplement: Supplementary file 2 — Supplementary Fig. S2. Rapamycin activates autophagy in MT‐COMP growth plate chondrocytes. Rapamycin reduces MT‐COMP retention in chondrocytes. Immunostaining with human COMP antibody of: (A) MT‐COMP growth plate chondrocytes at 4 weeks with no treatment, (B) control vehicle, (C) rapamycin (1 mg/kg 5 i.p. injections per week from 1 to 4 weeks of age). Ten mice were included in each treatment group. [file JBM4-5-e10456-s001.tif]
